# Supplementary material for: Colorectal Cancer Progression Is Potently Reduced by a Glucose-Free, High-Protein Diet: Comparison to Anti-EGFR Therapy
Source: Cancers (Basel). 2021 Nov 19;13(22):5817. doi: 10.3390/cancers13225817 (PMC8616508; doi:10.3390/cancers13225817)
Supplement: Supplementary file 1 [file cancers-13-05817-s001.zip › Figure S1.pdf]

Figure S1. Full Western blots.

Figure 3j

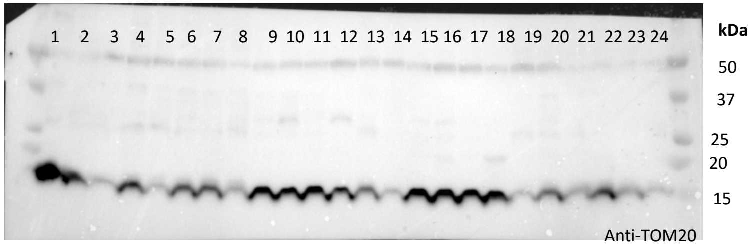

Figure 3h

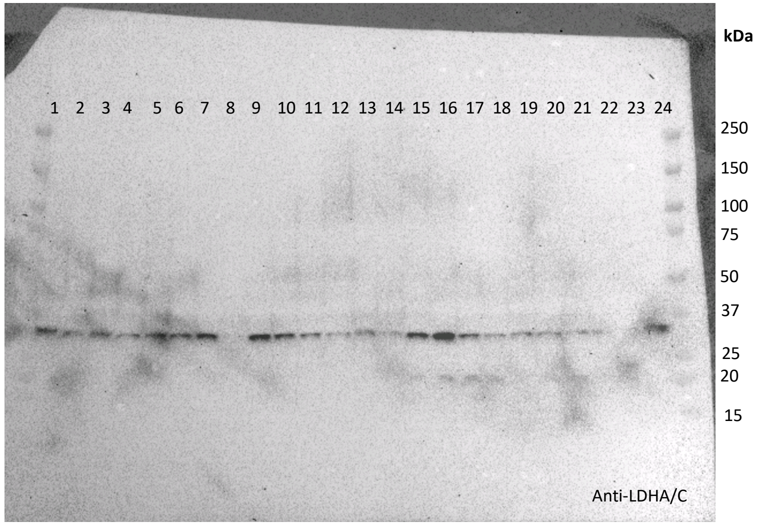

Figure 3i

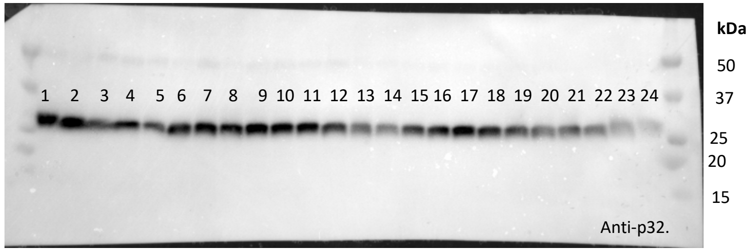

Figure 3m

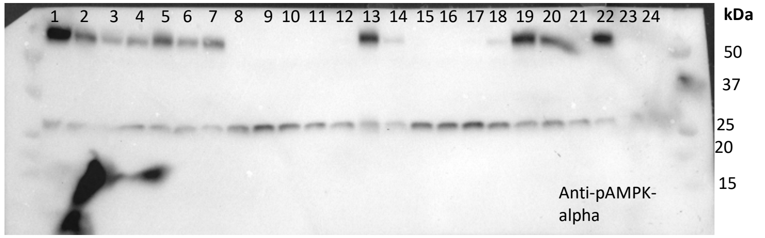

Figure 3h-j; m, n

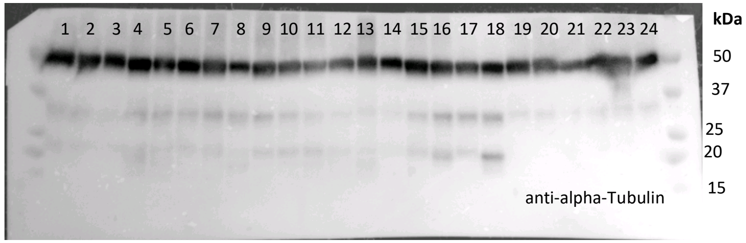

Figure 3n

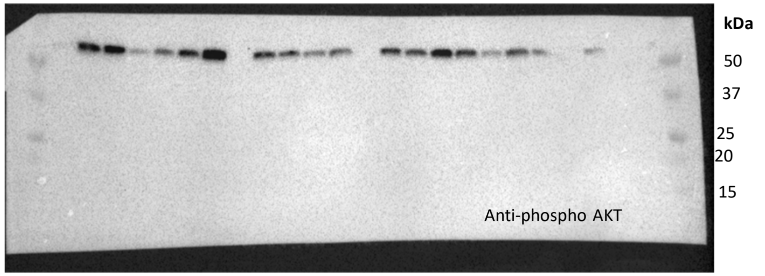

Figure 3k

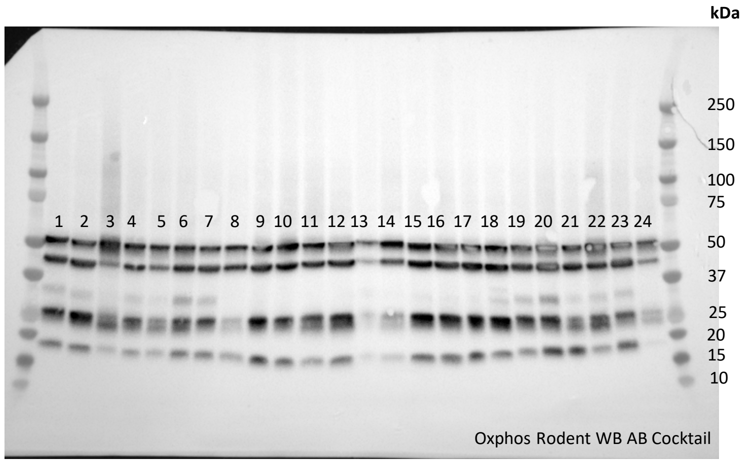
kDa
250
150
100
75
50
37
25
20
15
10
1 2 3 4 5 6 7 8 9 10 11 12 13 14 15 16 17 18 19 20 21 22 23 24
Oxphos Rodent WB AB Cocktail

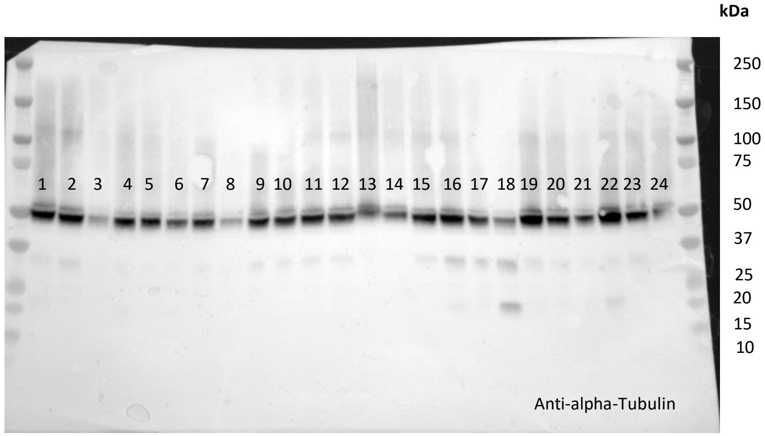
kDa
250
150
100
75
50
37
25
20
15
10
1 2 3 4 5 6 7 8 9 10 11 12 13 14 15 16 17 18 19 20 21 22 23 24
Anti-alpha-Tubulin

Figure 4k

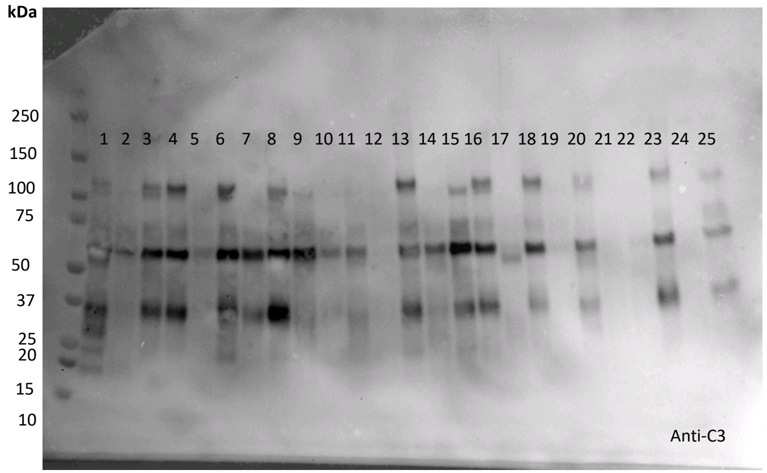
kDa
250
150
100
75
50
37
25
20
15
10
1 2 3 4 5 6 7 8 9 10 11 12 13 14 15 16 17 18 19 20 21 22 23 24 25
Anti-C3

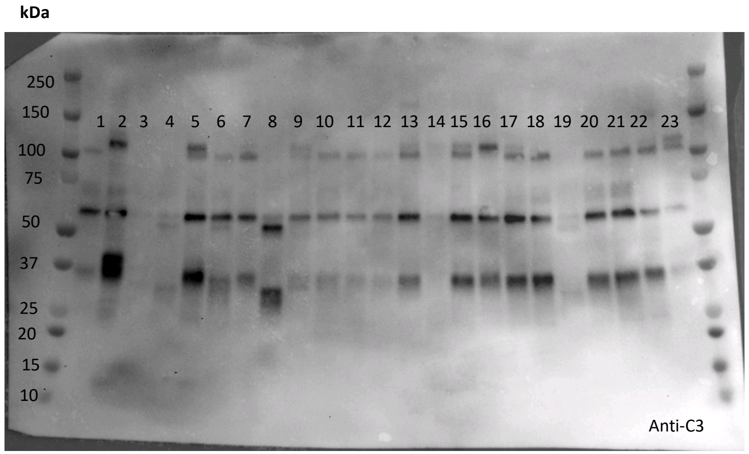
kDa
250
150
100
75
50
37
25
20
15
10
1 2 3 4 5 6 7 8 9 10 11 12 13 14 15 16 17 18 19 20 21 22 23
Anti-C3

Figure 5e

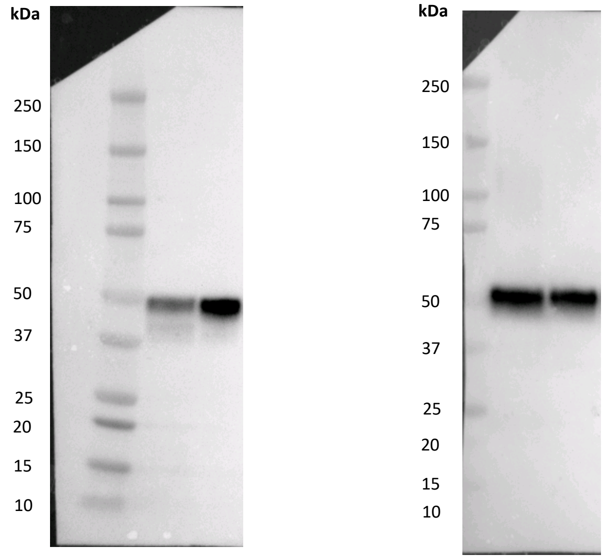

Anti-pro Caspase1

Anti-Alpha-Tubulin

Figure 5d

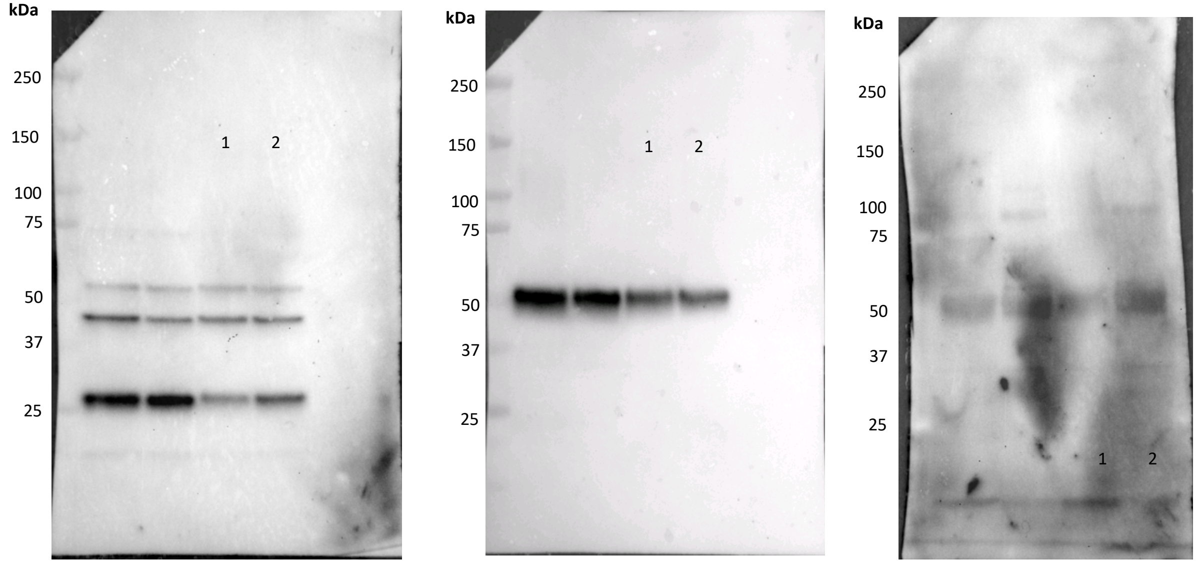

Oxphos Rodent Cocktail

Anti-Alpha-Tubulin

Anti-LC3B

Figure 5f

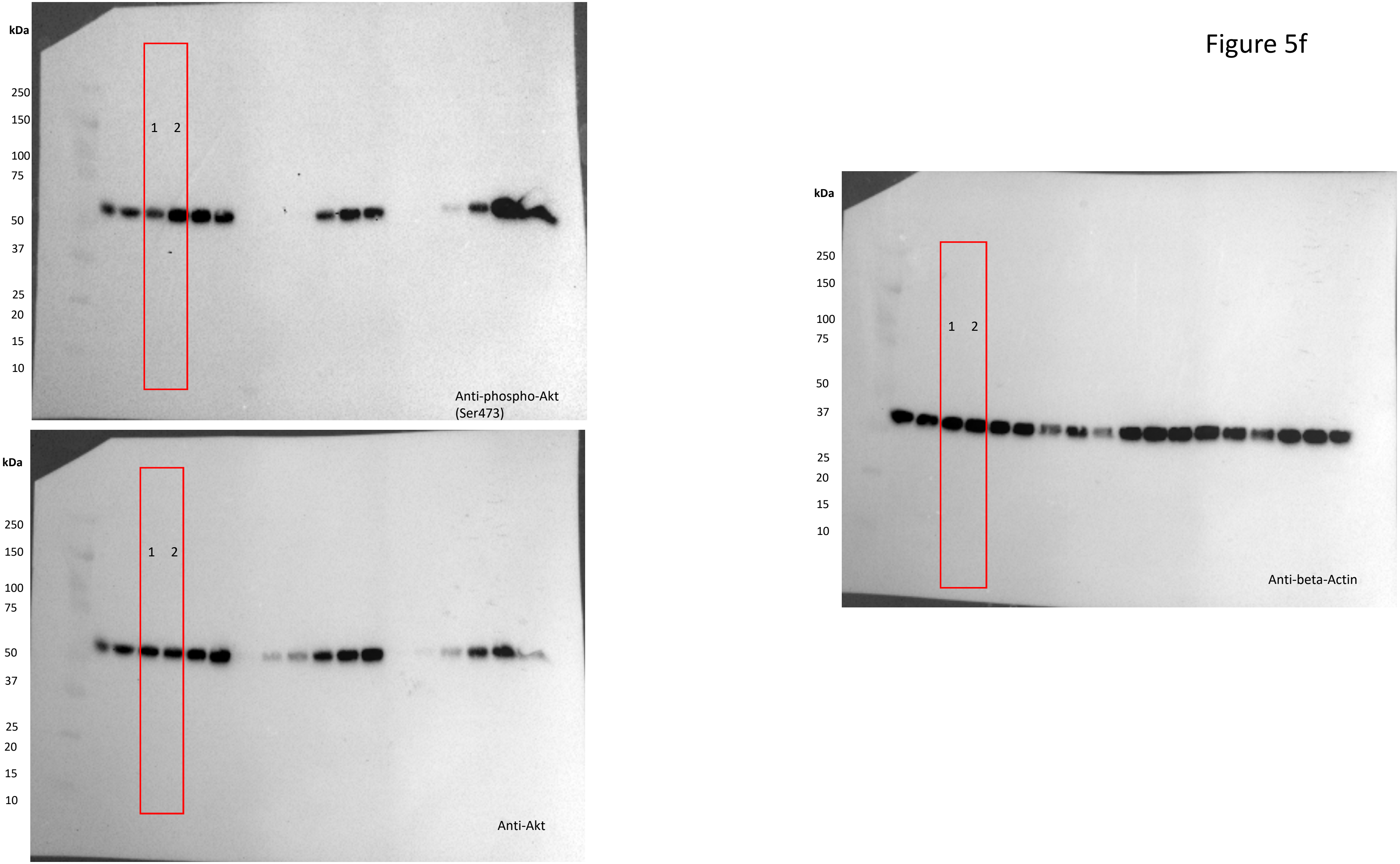

kDa
250
150
100
75
50
37
25
20
15
10
1 2
Anti-phospho-Akt
(Ser473)
kDa
250
150
100
75
50
37
25
20
15
10
1 2
Anti-Akt
kDa
250
150
100
75
50
37
25
20
15
10
1 2
Anti-beta-Actin

Figure 6f

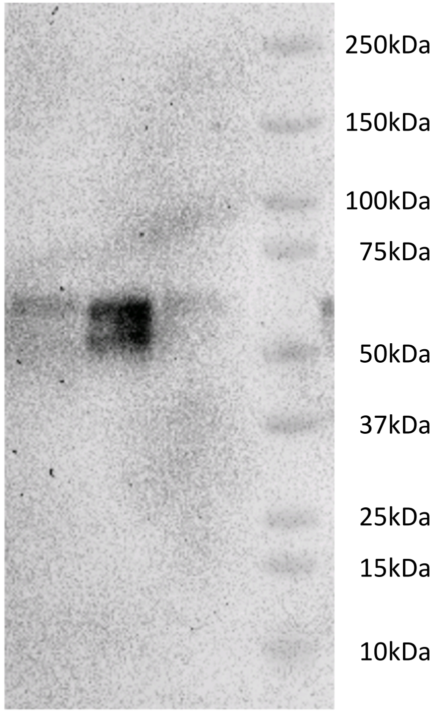

Anti-PD-L1

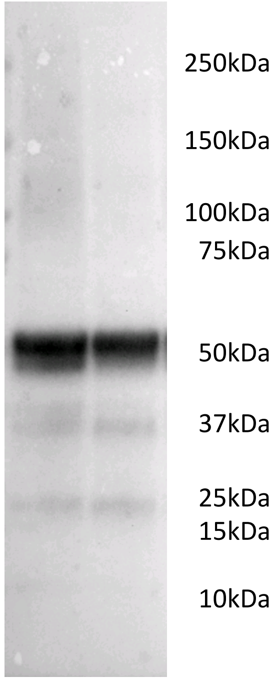

Anti-Alpha-Tubulin

Figure 6k

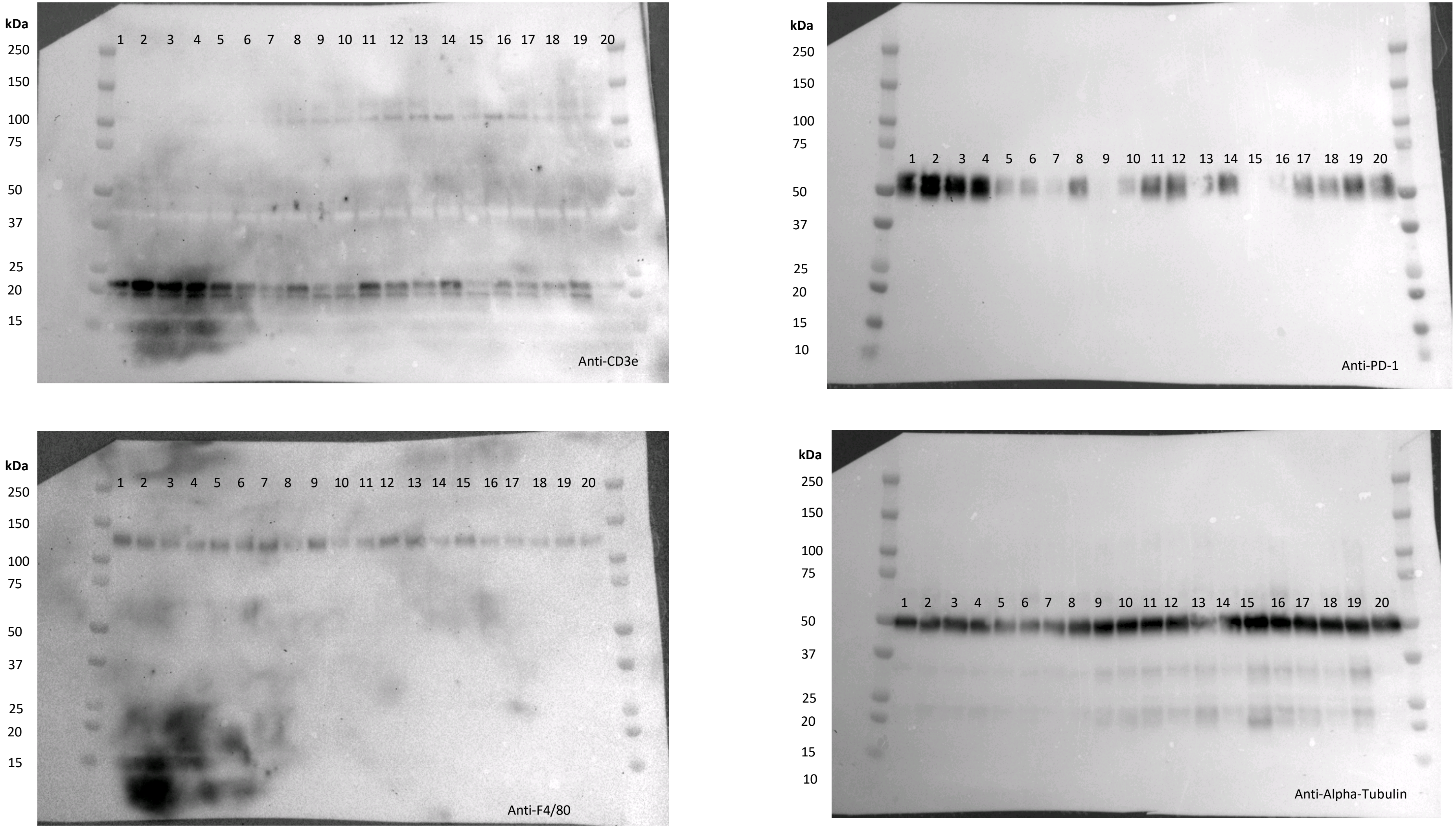
kDa
250
150
100
75
50
37
25
20
15
1 2 3 4 5 6 7 8 9 10 11 12 13 14 15 16 17 18 19 20
Anti-CD3e
kDa
250
150
100
75
50
37
25
20
15
10
1 2 3 4 5 6 7 8 9 10 11 12 13 14 15 16 17 18 19 20
Anti-PD-1
kDa
250
150
100
75
50
37
25
20
15
1 2 3 4 5 6 7 8 9 10 11 12 13 14 15 16 17 18 19 20
Anti-F4/80
kDa
250
150
100
75
50
37
25
20
15
10
1 2 3 4 5 6 7 8 9 10 11 12 13 14 15 16 17 18 19 20
Anti-Alpha-Tubulin

Figure 6l

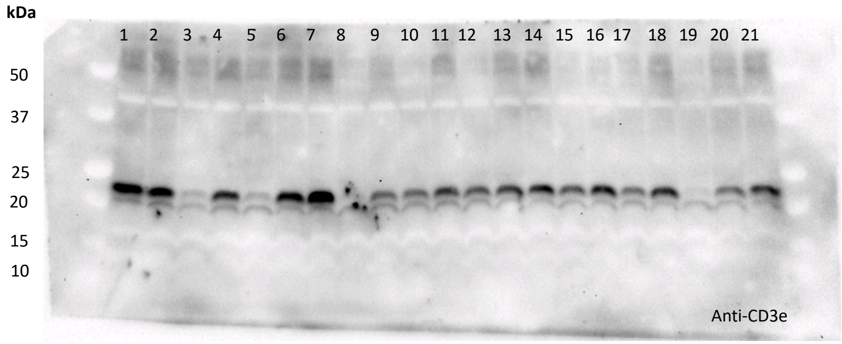
kDa
1 2 3 4 5 6 7 8 9 10 11 12 13 14 15 16 17 18 19 20 21
50
37
25
20
15
10
Anti-CD3e

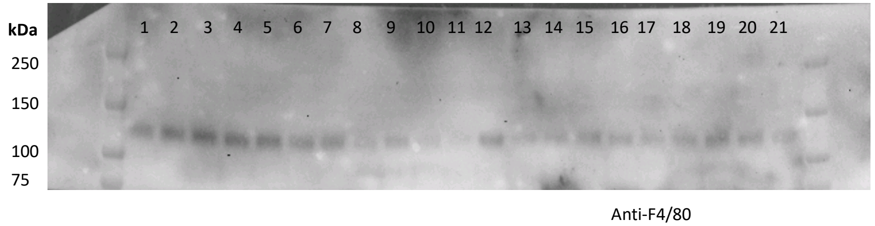
kDa
1 2 3 4 5 6 7 8 9 10 11 12 13 14 15 16 17 18 19 20 21
250
150
100
75
Anti-F4/80

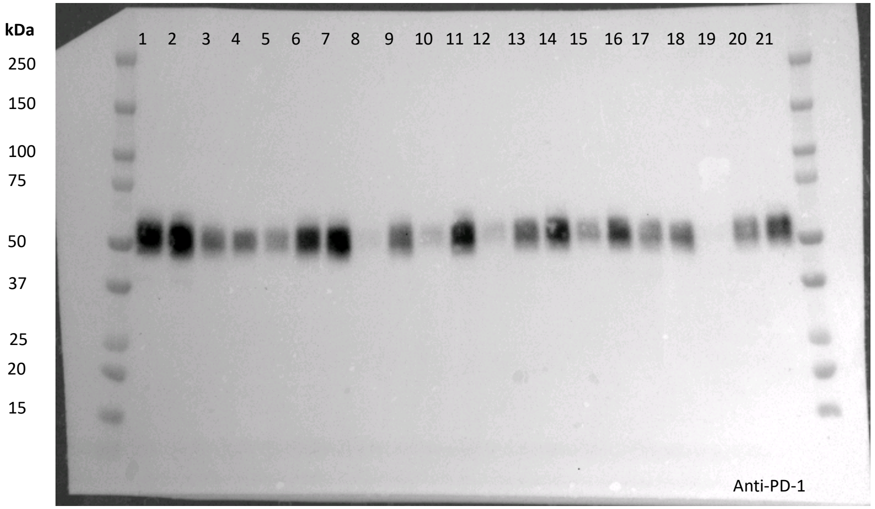
kDa
1 2 3 4 5 6 7 8 9 10 11 12 13 14 15 16 17 18 19 20 21
250
150
100
75
50
37
25
20
15
Anti-PD-1

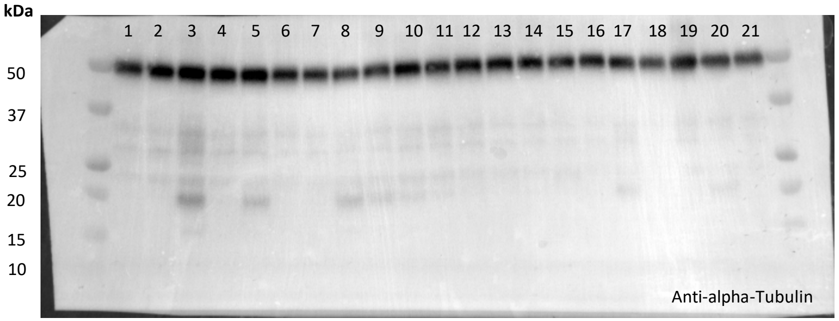
kDa
1 2 3 4 5 6 7 8 9 10 11 12 13 14 15 16 17 18 19 20 21
50
37
25
20
15
10
Anti-alpha-Tubulin
